# Biogeochemical niche conservatism relates to plant species diversification and life form evolution in a subtropical montane evergreen broad‐leaved forest

**DOI:** 10.1002/ece3.9587

**Published:** 2022-12-03

**Authors:** Kundong Bai, Xuewen Zhou, Shihong Lv, Shiguang Wei, Lili Deng, Yibo Tan

**Affiliations:** ^1^ Key Laboratory of Ecology of Rare and Endangered Species and Environmental Protection (Guangxi Normal University), Ministry of Education Guiling China; ^2^ Guangxi Key Laboratory of Landscape Resources Conservation and Sustainable Utilization in Lijiang River Basin Guangxi Normal University Guiling China; ^3^ Guangxi Lijiangyuan Forest Ecosystem Research Station Nanning China; ^4^ Guangxi Zhuang Autonomous Region and Chinese Academy of Sciences Guangxi Institute of Botany Guiling China; ^5^ Xing'an Guilin Lijiangyuan Forest Ecosystem Observation and Research Station of Guangxi Nanning China

**Keywords:** biogeochemical niche, conservatism, convergence, element interactions, life forms, Ornstein–Uhlenbeck process

## Abstract

The evolutionary mechanisms underlying the biogeochemical niche conservatism in forests remain incompletely understood. Here we aimed to determine how the strengths of biogeochemical niche conservatism vary among elements and between life forms. We measured leaf concentrations of basal elements (C, N, P, K, Ca, and Mg) in a wide range of life forms in a subtropical montane evergreen broad‐leaved forest. We found that differences in life forms such as evergreen/deciduous woody species and herbaceous/woody species significantly affected leaf elemental composition. The significant phylogenetic signal was present in leaf C, N, K, and Mg concentrations but absent in leaf P and Ca concentrations in all species. These contrasting strengths of biogeochemical niche conservatism were best generated by Ornstein–Uhlenbeck processes toward optima. Woody species were evolutionarily selected to show lower optimal leaf N, P, and K concentrations and higher optimal leaf C, Ca, and Mg concentrations than herbaceous species. The number of optima varied from the least in leaf C concentration to the most in leaf Ca concentration, suggesting the stronger convergent evolution of leaf Ca concentration. The positions of optima toward the tips were more selected in woody species, suggesting the more frequency of species‐specific adaptations in woody species. The positions of optima were also selected at the nodes towards the species groupings from certain life forms (e.g., the group of 12 Polypodiales ferns in leaf Ca evolution and the group of three evergreen Theaceae species in leaf P evolution) that were converged to present similar leaf elemental composition. During the evolution of biogeochemical niche, strong correlations were found among leaf C, N, P, and K concentrations and between leaf Ca and Mg concentrations. In conclusion, the strengths of biogeochemical niche conservatism can vary among elements and between life forms due to the different tempo and mode of Ornstein–Uhlenbeck processes.

## Graphical abstract

Our results show that biogeochemical niche conservatism relates to plant species diversification and life form evolution in subtropical forest plants. Furthermore, the strengths of biogeochemical niche conservatism varying among elements and between life forms might be mostly determined by the tempo and mode of OU processes.

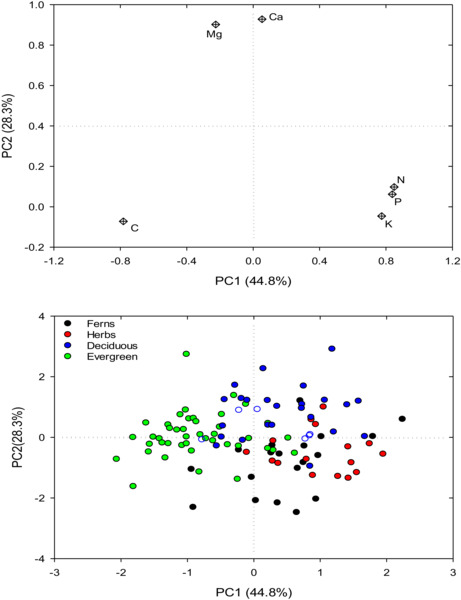

## 1 INTRODUCTION

Plant species vary greatly in the quantities of essential elements in leaves to survive and grow (Ågren, 2008; de la Riva et al., 2018). For example, the ranges of variation in leaf elemental concentrations were 2‐, 32‐, 75–108‐, 764‐, and 36‐fold for C, N, P, K, Ca, and Mg in global plant species, respectively (Ma et al., 2018; Metali et al., 2015). These contrasting concentration ranges of the six essential elements in leaves across plant species suggest that species generally have different requirements of essential elements in leaves and should thus have their own leaf elemental composition, which can be called biogeochemical niche (Peñuelas et al., 2008, 2019). Biogeochemical niches in plant species can be phylogenetically conserved (Fernández‐Martínez et al., 2021). Biogeochemical niche conservatism is usually estimated by the phylogenetic signal in leaf elemental composition, that is, the tendency for closely related species to show similar leaf elemental composition (e.g., Peñuelas et al., 2010; Sardans et al., 2021). Significant phylogenetic signal has been detected in leaf elemental composition in field‐collected plants (e.g., leaf C, N, P, K, and Mg concentrations in tropical and temperate forest plant species), lending support to biogeochemical niche conservatism (Duffin et al., 2019; Peñuelas et al., 2010). However, the evolutionary mechanisms generating biogeochemical niche conservatism in plant species remain incompletely understood (Fernández‐Martínez et al., 2021; Peñuelas et al., 2019).

Discerning the evolutionary mechanisms responsible for biogeochemical niche conservatism in plant species is a challenge that is complicated by three problems. First, biogeochemical niche conservatism can be generated by a variety of evolutionary processes. Cooper et al. (2010) have outlined that the patterns of niche conservatism can be underlain by five evolutionary models (drift, niche retention, niche filling/shifting, phylogenetic inertia, and evolutionary rate). These models describe that species niches have evolved through a pure Brownian motion (BM) process (i.e., drift model), a more expected BM process (i.e., niche retention model), a BM process with decreased/accelerated evolutionary rate (i.e., niche filling/shifting model), or a BM process with the incorporation of stabilizing selection (i.e., phylogenetic inertia model; Cooper et al., 2010). Previous studies have found that the values of Blomberg's *K* or Pagel's λ in leaf elemental concentrations are usually found to be lower than unity (i.e., pure BM process) even if they differ significantly from zero (i.e., no phylogenetic signal; e.g., Duffin et al., 2019; Hao et al., 2015; Peñuelas et al., 2010; Sardans et al., 2015), suggesting that the generation of biogeochemical niche conservatism might not follow a drift or niche retention model (Cooper et al., 2010). Several studies have shown that the values of Pagel's δ were higher than unity for leaf N, P, K, Ca, Mg, S, Fe, and Zn concentrations in tropical karst tree species and cave‐dwelling herbaceous species (Bai et al., 2019, 2020), suggesting that biogeochemical niche conservatism could be generated by niche shifting model (Cooper et al., 2010; Pagel, 1999). Alternatively, some studies have shown that the generation of biogeochemical niche conservatism might follow the phylogenetic inertia model, that is, stabilizing selection toward an optimum or optima (Bai et al., 2019, 2020; Fernández‐Martínez et al., 2018). For example, Fernández‐Martínez et al. (2018) found that the evolution of leaf P concentration could be best described by stabilizing selection towards an optimum in global plants. In contrast, they found that the higher values of optimal leaf N concentration were evolutionarily selected in some families such as Betulaceae and Salicaceae. In addition, a high evolutionary rate of change in one group compared with another is evidence of less‐constrained evolution for the same trait in two groups (i.e., evolutionary rate model; Cooper et al., 2010). This is the case in biogeochemical niche conservatism. For instance, Bai et al. (2020) found the lower evolutionary rates of leaf N, P, K, Ca, Mg, S, Fe, Mn, and Zn concentrations in ferns than herbs in caves. Since the generation of biogeochemical niche conservatism has been supported by different evolutionary models, researchers can compare which evolutionary model best fits dataset (Cooper et al., 2010; Münkemüller et al., 2015).

Second, the strength of biogeochemical niche conservatism might vary among elements. Previous studies have often identified that phylogenetic signals were present in some leaf elements but absent in other leaf elements in an environment. For example, Sardans et al. (2015) have detected that phylogenetic signal was significant in leaf N, K, Ca, Mg, and S concentrations but weak in leaf P concentration in European forest tree species. The contrasting levels of phylogenetic signals among leaf elements might be related to their different evolutionary rate and processes. This is supported by the finding that phylogenetic signal was strongly and negatively correlated with evolutionary rate and the strengths of niche shifting and phylogenetic inertia across leaf N, P, K, Ca, Mg, S, Fe Cu, and Zn concentrations in tropical karst tree species (Bai et al., 2019). The contrasting levels of phylogenetic signals among leaf elements might also be related to their different nutritional statuses. For example, the generally observed P limitation in tropical and subtropical soils could make distantly related species to present similar and low leaf P concentration and thus erase the phylogenetic signal in leaf P concentration (Bai et al., 2019; Metali et al., 2012; Wen et al., 2018). In addition, the evolution of leaf elements could be coordinated. A typical example is that leaf Ca and Mg concentrations are commonly observed to be positively interacted among specific groups of angiosperms when their growth is not limited by mineral nutrition, indicating that a significant historical relationship between the two elements exists throughout the evolutionary history of angiosperms (Hernández et al., 2013; Neugebauer et al., 2018). This could partly explain why some elements such as Ca and Mg present significant phylogenetic signals at the same time. Collectively, the strength of biogeochemical niche conservatism should be evaluated with the considerations of individual element evolution and coordinated evolution among elements.

Finally, the strength of biogeochemical niche conservatism might be different between plant life forms. Life forms refer to groups of plant species with similar morphology (e.g., woody/herbaceous species, herbs/ferns) and phenology (e.g., evergreen/deciduous woody species; Watanabe & Azuma, 2021). Recent research progresses have provided evidence for the different biogeochemical niche conservatism between life forms. One is from the detection of phylogenetic signal. For instance, Laine et al. (2021) have found that the phylogenetic signals in leaf C and N concentrations were absent in Sphagnum species but present in vascular plants. The other is from the detection of evolutionary rate and evolutionary process. For example, Bai et al. (2020) have demonstrated that herbs tend to have higher evolutionary rate than ferns for leaf N, P, K, Ca, Mg, S, Fe, Mn, and Zn concentrations in caves. Moreover, they found that the higher optimal values of leaf Ca, N, and Mg were evolutionary selected in cave‐dwelling herbs. Therefore, we predicted that the contrasting strengths of biogeochemical niche conservatism between life forms might be related to their different evolutionary rates and processes in an environment.

The triptych of problems suggests the necessity to choose the best‐fitted evolutionary process to evaluate the strengths of biogeochemical niche conservatism among elements and between life forms. To our knowledge, little attention has been paid to determine the evolutionary processes responsible for the contrasting biogeochemical niche conservatism among elements and between life forms. In this work, our goal was thus to determine how the strength of biogeochemical niche conservatism differs among elements and between life forms. To address this goal, we measured leaf concentrations of basal elements (C, N, P, K, Ca, and Mg) in a wide range of herbaceous and woody species in a subtropical montane evergreen broad‐leaved forest in southern China (Bai et al., 2015). Similar to the typical lowland tropical forests, the soil in this subtropical forest is highly leached due to the strong mean annual precipitation (i.e., >2500 mm) and thus tends to be P‐limited (Huang & Jiang, 2002; Spicer et al., 2020). However, this subtropical forest is species‐rich and contains more woody species than herbaceous species (Huang & Jiang, 2002; Spicer et al., 2020). Within herbaceous species concentrated on the forest floor, ferns are more abundant than herbs. Within woody species dominating the forest understory and canopy layers, there also exists the variation in species richness due to leaf habits. Evergreen lineages such as Fagaceae are more abundant than deciduous lineages such as Sapindaceae (Huang & Jiang, 2002). The P limitation in soil and contrasting species abundance patterns between life forms, together with the generally observed biogeochemical niche conservatism, make it reasonable for us to test the idea that biogeochemical niche conservatism relates to plant species diversification and life form evolution. Specifically, we asked two questions: (1) does biogeochemical niche differ between life forms (i.e., ferns/herbs, evergreen/deciduous woody species, and herbaceous/woody species)?; (2) does the strength of biogeochemical niche conservatism differ among elements and between life forms?

## 2 MATERIALS AND METHODS

### 2.1 Site selection

**FIGURE A1:**
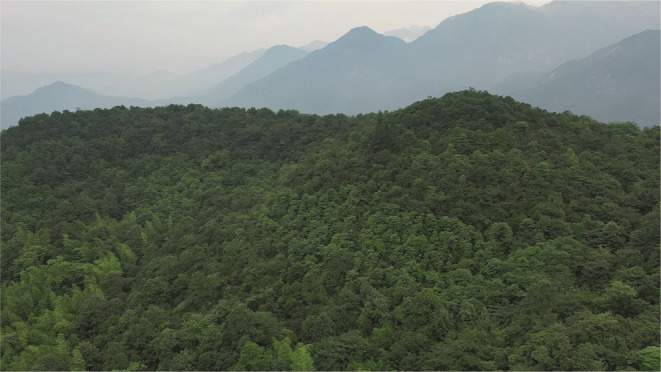
An overview on our sample subtropical montane evergreen broad‐leaved forest.

We selected a subtropical evergreen broad‐leaved forest at altitudes between 950 and 1200 m in Guangxi Mao'er Mountain National Reserve (25°50′N, 110°49′E), southern China (Bai et al., 2015; Figure A1 in Appendix). The forest is primarily old‐growth but has disturbed parts due to natural disasters such as winter ice storm and human disturbances such as ecological tourism. The forest is mainly dominated by *Castanopsis fabri*, *C. fargesii*, *C. caresii*, *Schima superba*, and *Rhododendron elliptic* in the forest canopy and *Diplopterygium chinese*, *Woodwardia japonica*, and *Lophatherum gracile* in the forest floor. Soil substrate in the forest is montane yellow soil. Soil pH, concentrations of organic matter, N, P and K, and cation exchange capacity are 4.57%, 14%, 0.5%, 0.12%, 2.1% and 28.7 me/100 g, respectively (Huang & Jiang, 2002). According to the climatic observations conducted by Guangxi Lijiangyuan Forest Ecosystem Research Station near to the forest (i.e., at 1100 m altitude in Mao'er Mountain), mean annual temperature and mean annual precipitation from 2016 to 2020 were 14.0°C and 3342 mm, respectively.

### 2.2 Plant sampling and measurement

We sampled the co‐existing herbaceous and woody species in the forest in July 2019 and 2020. The sampled herbaceous species included 19 ferns and 16 herbs. Among woody species, the number of evergreen and deciduous species was 43 and 24, respectively. Collectively, the total 102 plant species were from 77 genera and 45 families (Table A1 in Appendix).

To quantify leaf elemental concentrations, we collected leaf samples using a unified protocol (Zhao et al., 2016). For herbaceous species, we sampled a certain number of individuals per species that could provide enough leaf samples for measurement. For woody species, we sampled the current year's fully expanded and canopy leaves from three individuals per species. The harvested leaf samples of each species were oven‐dried at 60°C for 72 h and ground to fine powder for chemical analyses. Each species had three measurement replicates. To determine leaf C and N concentrations, leaf samples were analyzed using an elemental analyzer (Vario MAX CN Elemental Analyzer). Leaf P, K, Ca, and Mg concentrations were determined using an inductively coupled plasma emission spectrum (iCAP Qc, Thermo Fisher Scientific; Bai et al., 2020).

### 2.3 Data analysis

#### 2.3.1 Testing for biogeochemical niche differences in plants

To test biogeochemical niche differences between life forms (i.e., ferns/herbs, evergreen/deciduous woody species, and herbaceous/woody species), we first performed analyses of variance (ANOVA) for each element using log_10_‐transformed leaf elemental concentrations. In addition to univariate element, we also investigated the evolution of biogeochemical niche in a multivariate sense, using a principal component analysis (PCA) on mean leaf elemental concentrations across all species (Sardans et al., 2021). The first two PCA axes explained a large proportion of total variation in leaf elemental composition. We then performed ANOVAs to detect whether the species scores of the first two PCA axes (PC1 and PC2) differed between life forms. The above conventional statistical analyses were conducted in SPSS 13.0 (SPSS).

#### 2.3.2 Testing for biogeochemical niche conservatism in plants

**FIGURE 1:**
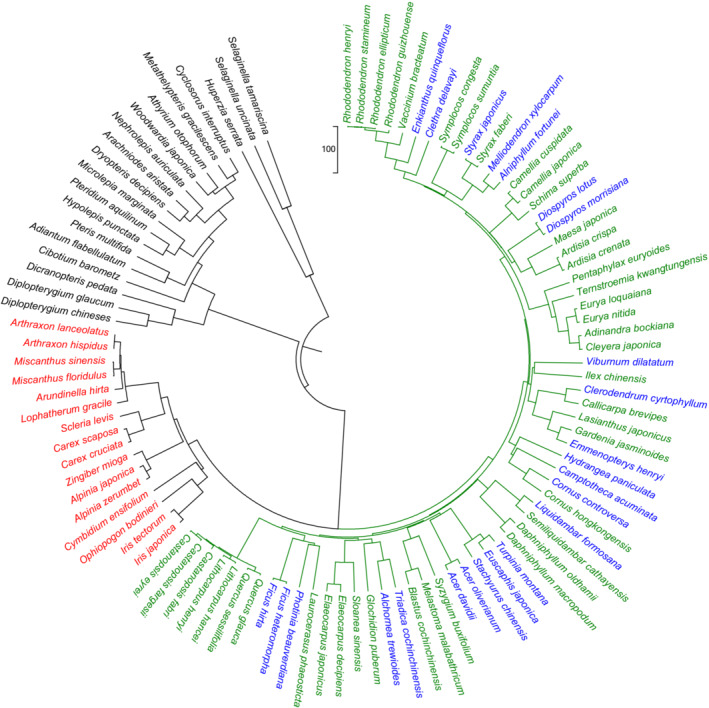
Phylogenetic relationships among the co‐existing herbaceous (ferns in black, herbs in red) and woody (deciduous in blue, evergreen in green) species in a subtropical montane evergreen broad‐leaved forest.

We constructed a phylogenetic tree of the sampled species using the V. PhyloMaker package in R (Jin & Qian, 2019) that generates phylogenies for plants based on the backbone mega‐tree of 10,587 genera and 74,533 species of vascular plants, that is, GBOTB (Smith & Brown, 2018; Zanne et al., 2014). All 45 families in our study were included in GBOTB and thus were well resolved at the family level. Of the 102 species in our study, 84 species were matched with those in GBOTB. For the remaining 18 species (i.e., *C. fabri*, *R. guizhouense*, *Elaeocarpus decipiens*, *Callicarpa brevipes*, *Laurocerasus phaeosticta*, *Daphniphyllum oldhamii*, *Scleria levis*, *Miscanthus floridulus*, *Huperzia serrata*, *Athyrium otophorum*, *W. japonica*, *Arachniodes aristate*, *Dryopteris decipiens*, *D. chineses*, *Nephrolepis auriculata*, *Cyclosorus interruptus*, *Metathelypteris gracilescens*, and *Selaginella uncinata*) that were missing from GBOTB, they were treated as sisters and added randomly to their closely related genera in GBOTB (i.e., scenario 2 in V. PhyloMaker; Jin & Qian, 2019). We finally got a tree with distinct life forms and time‐calibrated branch lengths for the subsequent phylogenetic analyses (Figure 1). Like previous studies (e.g., Zhang et al., 2021, 2022), the insertion of the 18 species into the backbone tree could lead to phylogenetic uncertainty. To account for such phylogenetic uncertainty, we generated 100 random trees by scenario 2 and used them to replicate phylogenetic analyses to evaluate the stability of biogeochemical niche conservatism pattern during species diversification (Table A1 in Appendix).

To compare the strengths of biogeochemical niche conservatism among elements and between life forms, we conducted a series of phylogenetic analyses in R (version 4.1.2; R Development Core Team, 2008). Before phylogenetic analyses, species mean values of leaf elemental concentrations were log_10_‐transformed and tree depth was scaled to unity via the rescale function in the GEIGER package (Harmon et al., 2008). We first detected the phylogenetic signals in leaf elemental concentrations and PC1 and PC2 species scores using Blomberg's *K* statistic in all species to get the general patterns of biogeochemical niche variation during species diversification (Blomberg et al., 2003). The Blomberg's *K* values were tested for the null hypothesis of absence of the phylogenetic signal, that is, trait values randomly distributed in the phylogenetic tree. This was achieved by randomization for *K* (999 times). The *K* values significantly larger than zero suggest significant phylogenetic signals. The *K* values equal to unity suggest traits have evolved following BM process (i.e., drift model) and *K* values larger than unity indicate closely related species more similar than expected under BM process (i.e., niche retention model; Cooper et al., 2010). We used the phyloSignal function in the PHYLOSIGNAL package to detect phylogenetic signals (Keck et al., 2016).

We then compared a set of evolutionary models fitting to leaf elemental concentrations and PC1 and PC2 species scores in all species. Models included BM, Pagel's δ and Ornstein–Uhlenbeck (OU). BM is a neutral model of evolution where species traits have evolved via random‐walk at a constant rate (i.e., sigma). We fitted the BM models with a single evolutionary rate and two different evolutionary rates for herbaceous and woody species (BMM). We fitted BMM because the two life forms grew in contrasting light environments. Herbaceous species were concentrated on the forest floor where low light availability might condition the evolution of biogeochemical niche. We fitted BM and BMM using the mvBM function in the mvMORPH package (Clavel et al., 2015). Pagel's δ describes that species trait evolution has accelerated or slowed down over time. The values of δ lower than unity indicate temporally early trait evolution or early‐burst (i.e., niche filling model) and the values of δ larger than unity indicate temporally latter trait evolution (i.e., niche shifting model). We fitted Pagel's δ model via the fitContinuous function in the GEIGER package (Harmon et al., 2008). We tested whether δ was significantly different from unity using the pgls function in the CAPER package (Orme et al., 2012). OU describes that species traits have evolved toward an optimum or optima under a stabilizing strength (i.e., phylogenetic inertia, ɑ). We fitted the OU model with a single optimum for all species (OU1) and two optima (OUM) to test whether selective optima differed between herbaceous and woody species. We fitted OU1 and OUM using the mvOU function in the mvMORPH package. To evaluate the possible different optima within herbaceous or woody species in the phylogeny, we detected the positions of optima in the phylogeny using the OUshifts function with the singular Bayesian information criterion penalty in the PHYLOLM package (Tung Ho & Ané, 2014). Comparisons of BM, BMM, Pagel's δ, OU1, and OUM models were based on the sample size‐corrected Akaike information criterion (AICc; e.g., Bai et al., 2020; Fernández‐Martínez et al., 2018). When models differed by ΔAICc < 2, they were considered approximately equivalent (Burnham & Anderson, 2002), and the model with the lowest AICc value was considered the best‐fitted.

To test the coordinated evolution among elements, we finally explored correlations between leaf elemental concentrations in all species by phylogenetic generalized least squares with a phylogenetic covariance matrix, simultaneously estimating Pagel's λ for phylogenetic heritability of regression residues (McCormack et al., 2020; Pagel, 1999). We used the pgls function in the CAPER package to get the element interaction strengths (Orme et al., 2012).

## 3 RESULTS

### 3.1 Biogeochemical niche differences in plants

**FIGURE 2:**
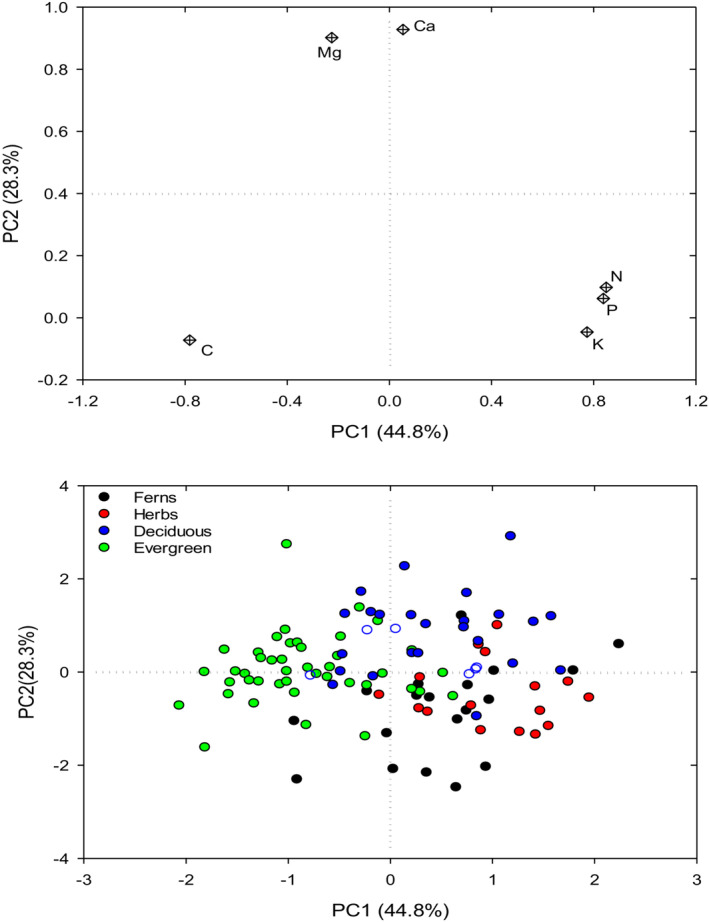
Principal component analysis for the six leaf elemental concentrations and species scores of the first two axes (PC1 and PC2) across herbaceous (ferns in black, herbs in red) and woody (deciduous in blue, evergreen in green) species are shown.

Mean leaf elemental concentrations across all species varied from 1.61 mg g^−1^ for P to 411 mg g^−1^ for C (Table 1). The ranges of leaf C, N, P, K, Ca, and Mg concentrations across all species were 1.6‐, 2.7‐, 4.7‐, 5.3‐, 31.3‐, and 7.6‐fold, respectively (Table A2 in Appendix). Leaf elemental composition significantly differed between herbaceous and woody species, which was characterized by higher leaf N, P, and K concentrations but lower leaf C, Ca, and Mg concentration in herbaceous species (Table 1). Within herbaceous species, ferns tended to have lower leaf N and P concentrations than herbs (Table 1). Within woody species, leaf elemental composition was significantly affected by leaf habit (Table 1). Evergreen woody species had lower leaf N, P, K, Ca, and Mg concentrations but higher leaf C concentrations than deciduous woody species (Table 1). The PCA showed that the first two axes (i.e., PC1 and PC2) explained accumulative 73.1% of the total variation in leaf elemental composition (Figure 2). Leaf C concentration loaded negatively and leaf N, P, and K concentrations positively with PC1 and leaf Ca and Mg concentrations loaded positively with PC2 (Figure 2). The PC1 and PC2 species scores differed significantly between herbaceous and woody species and between evergreen and deciduous woody species (Table 1), again suggesting the biogeochemical niche variations between herbaceous and woody species and between leaf habits (Figure 2).

**TABLE 1:** Summary of analysis of variance for the comparisons of leaf elemental concentrations (mean ± standard error; mg g^−1^) and species scores of the first two principal component analysis axes (PC1 and PC2) between life forms.

Life form	*n*	C	N	P	K	Ca	Mg	PC1	PC2
All species	102	411 ± 3	22.21 ± 0.49	1.61 ± 0.05	12.89 ± 0.49	9.02 ± 0.58	3.49 ± 0.16	0.000 ± 0.099	−0.000 ± 0.099
Woody species	67	420 ± 3A	21.11 ± 0.57A	1.50 ± 0.06A	10.82 ± 0.51A	10.18 ± 0.76A	4.12 ± 0.20A	−0.385 ± 0.110A	0.354 ± 0.106A
Evergreen	43	433 ± 4a	19.16 ± 0.45a	1.30 ± 0.06a	9.08 ± 0.35a	8.49 ± 0.82a	3.73 ± 0.21a	−0.843 ± 0.099a	0.063 ± 0.112a
Deciduous	24	398 ± 4b	24.59 ± 1.07b	1.86 ± 0.10b	13.94 ± 1.01b	13.20 ± 1.37b	4.81 ± 0.40b	0.436 ± 0.142b	0.876 ± 0.174b
Herbaceous species	35	393 ± 5B	24.31 ± 0.82B	1.81 ± 0.06B	16.84 ± 0.66B	6.81 ± 0.69B	2.30 ± 0.11B	0.737 ± 0.124B	−0.678 ± 0.147B
Ferns	19	396 ± 9a	22.44 ± 1.07a	1.69 ± 0.07a	16.09 ± 0.88a	6.58 ± 1.06a	2.23 ± 0.15a	0.507 ± 0.178a	−0.838 ± 0.233a
Herbs	16	390 ± 5a	26.53 ± 1.03b	1.94 ± 0.08b	17.73 ± 0.96a	7.08 ± 0.85a	2.38 ± 0.17a	1.010 ± 0.146b	−0.488 ± 0.174a

*Note*: *n* is the number of species. The different upper case letters indicate significant differences between herbaceous and woody species. The different lower case letters indicate significant differences between evergreen and deciduous woody species or between ferns and herbs.

### 3.2 Biogeochemical niche conservatism in plants

The phylogenetic signals in leaf elemental composition (i.e., Blomberg's *K*) varied from 0.056 (i.e., P) to 0.245 (i.e., C) in all species (Table 2). Leaf C, N, K, and Mg concentrations had significant phylogenetic signals and leaf Ca and P concentrations exhibited non‐significant phylogenetic signals. However, the phylogenetic signals in PC1 and PC2 species scores were all significant (Table 2). Our replicated phylogenetic signal analyses on 100 random trees suggested that the frequencies of trees showing similar results to the tree in Figure 1 were 96%, 100%, 97%, 92%, 98%, 81%, 100%, 80% in leaf C, N, P, K, Ca, and Mg concentrations and PC1 and PC2 species scores, respectively (Table A1 in Appendix), indicating the highly stable pattern in biogeochemical niche conservatism during species diversification.

**TABLE 2:** Values of phylogenetic signal (Blomberg's *K*) and the comparisons of evolutionary models fitting to leaf elemental concentrations and species scores of the first two principal component analysis axes (PC1 and PC2) across all species.

Variable	*K*	BM		BMM			OU1			OUM			Pagel's δ	
		AICc	Sigma	AICc	Sigma_H_	Sigma_W_	AICc	ɑ	Opt	AICc	Opt_H_	Opt_W_	AICc	δ
C	0.245**	−351.94	0.013	−362.16	0.006	0.017	−417.37	23.35	407	−427.02	393	416	−417.27	46.74
N	0.090**	−117.40	0.130	−126.15	0.059	0.167	−198.48	30.04	21.74	−202.06	23.44	20.85	−198.37	60.69
P	0.056	21.14	0.505	−7.72	0.110	0.714	−112.26	233.03	1.54	−120.97	1.77	1.43	−112.26	467.08
K	0.117*	6.18	0.436	3.36	0.266	0.527	−71.40	25.29	12.28	−96.45	16.20	10.37	−71.26	50.91
Ca	0.071	119.32	1.322	112.37	0.657	1.676	26.98	188.54	7.63	15.97	5.65	8.93	26.98	379.90
Mg	0.101**	13.05	0.466	4.43	0.213	0.602	−55.74	63.31	3.21	−105.17	2.22	3.87	−55.71	131.83
PC1	0.146**	354.37	13.243	340.99	4.991	17.655	276.52	23.20	0.060	256.16	0.686	−0.316	276.72	46.74
PC2	0.073*	381.52	17.281	355.62	4.135	24.281	294.18	190.10	0.019	267.39	−0.675	0.376	294.19	381.94

*Note*: Significant levels of Blomber's *K* are marked: **p < .01, *p < .05. BM is the Brownian motion model with a single rate through the phylogeny and sigma is the evolutionary rate under the BM. BMM is the BM model with different evolutionary rates for herbacerous (sigma_H_) and woody (sigma_W_) species. OU1 is the Ornstein–Uhlenbeck model with a single optimum (Opt) and ɑ is the strength of stabilizing selection. OUM is the OU model with different optima for herbacerous (Opt_H_) and woody (Opt_W_) species. Pagel's δ is the degree of slowness or acceleration of trait evolution through the phylogeny. All δ values are significantly larger than unity in the examined variables. AICc is the sample‐size corrected Akaike information criterion.

Among the evolutionary processes fitting to leaf elemental concentrations and PC1 and PC2 species scores in all species, BM models were least supported due to the highest AICc values (Table 2). The parameter representing evolutionary rate under BM model (i.e., sigma) varied from 0.013 in leaf C concentration to 1.322 in leaf Ca concentration. Similarly, PC1 had lower sigma value than PC2. The BMM model outperformed BM model due to their ΔAICc differences larger than 2 (Table 2). The sigma values of leaf elemental concentrations and PC1 and PC2 scores in herbaceous species were lower than those in woody species.

Pagel's δ models were also better than BM models (Table 2). The δ values were all significantly higher than unity and varied from 46.74 in leaf C concentration to 467.08 in leaf P concentration. OU1 models did not outperform Pagel's δ models due to their small ΔAICc differences (Table 2). But OU1 models outperformed BM models (Table 2). The strength of phylogenetic inertia (i.e., ɑ) ranged from 23.35 in leaf C concentration to 233.03 in leaf P concentration (Table 2). The δ and ɑ values in PC1 were lower than those in PC2 species. OUM models were the best models due to their lowest AICc values in leaf elemental concentrations and PC1 and PC2 species scores (Table 2). Herbaceous species had higher optimal values of leaf N, P, and K concentrations than woody species, which in turn had higher optimal values of leaf C, Ca, and Mg concentrations.

**FIGURE 3:**
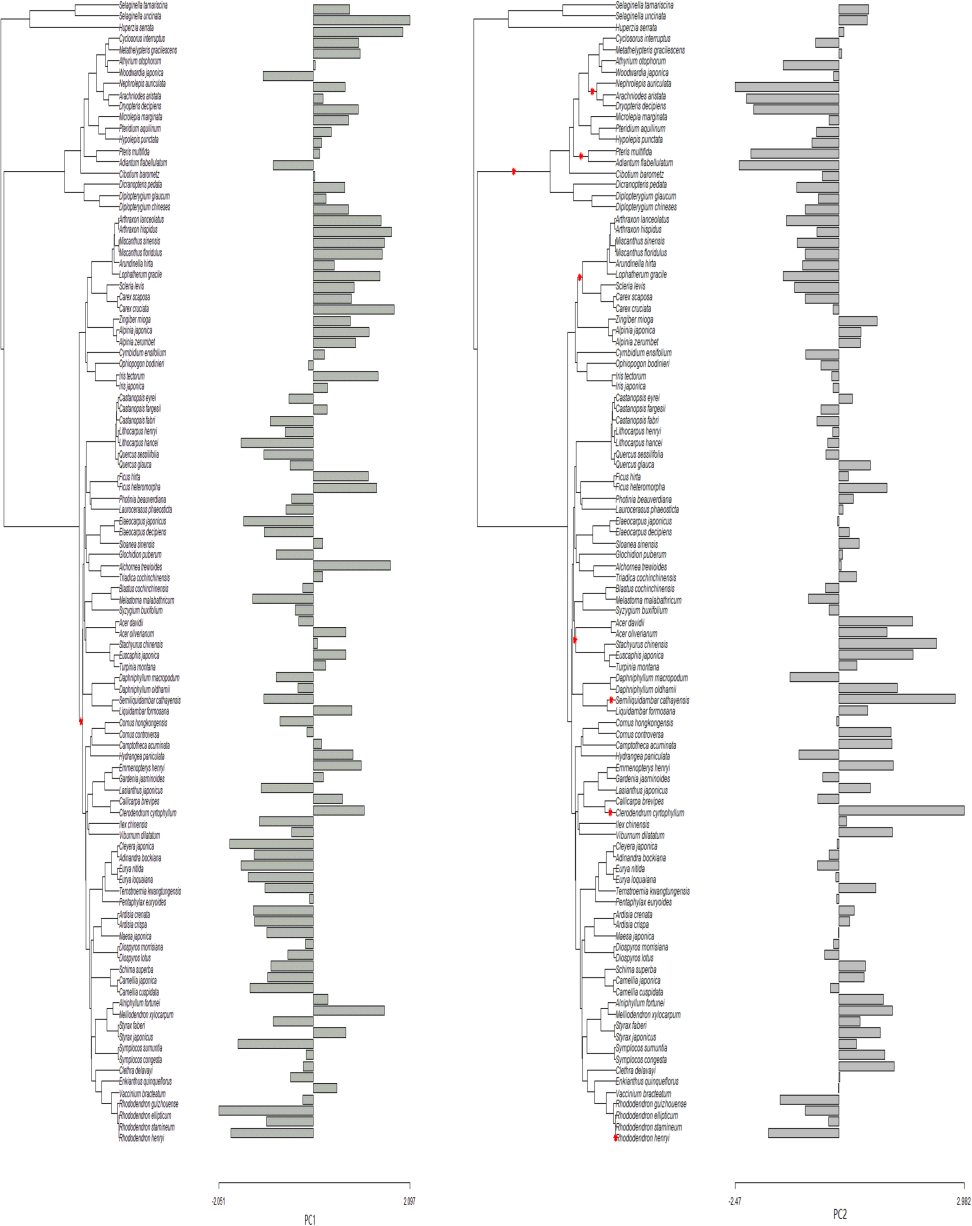
The positions (red stars) of the selective optima of species scores of the first two principal component axes (PC1 and PC2) in the phylogeny.

**FIGURE A2:**
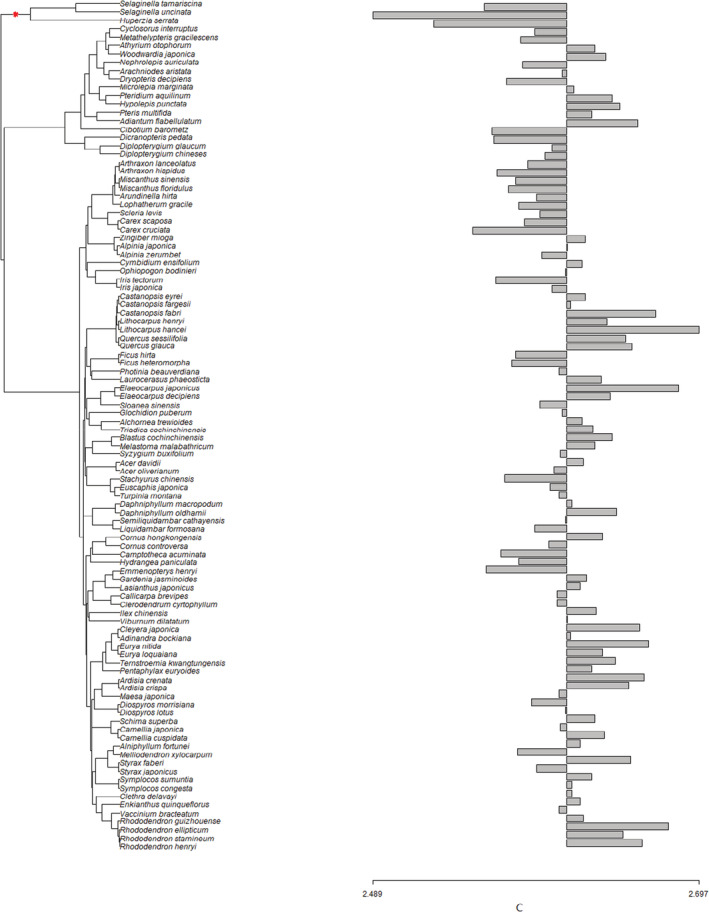
The positions (red stars) of the selective optima of leaf C concentration in the phylogeny.

**FIGURE A3:**
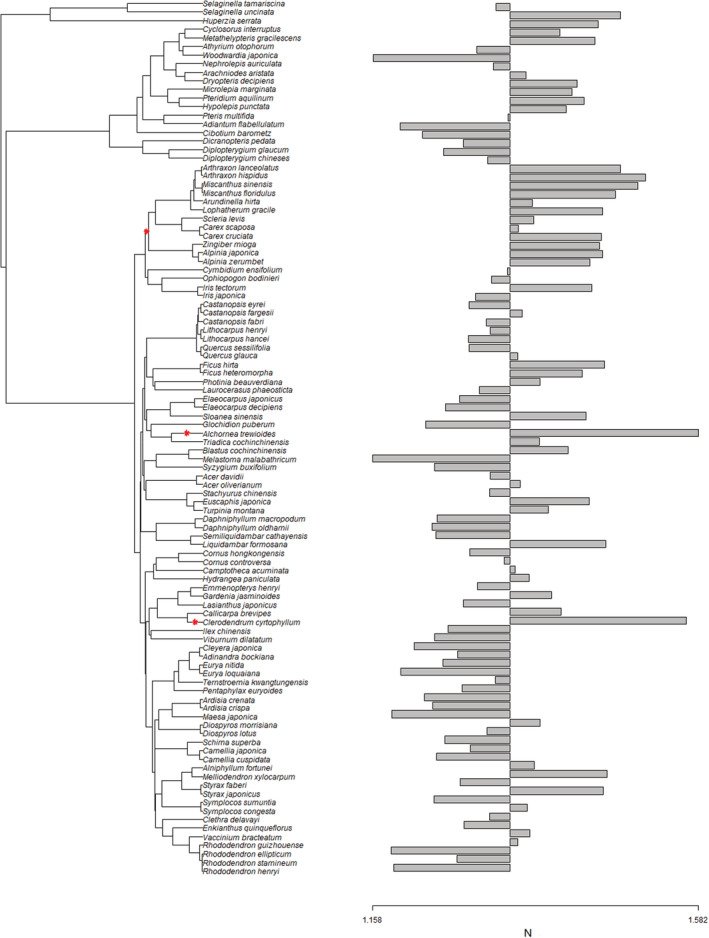
The positions (red stars) of the selective optima of leaf N concentration in the phylogeny.

**FIGURE A4:**
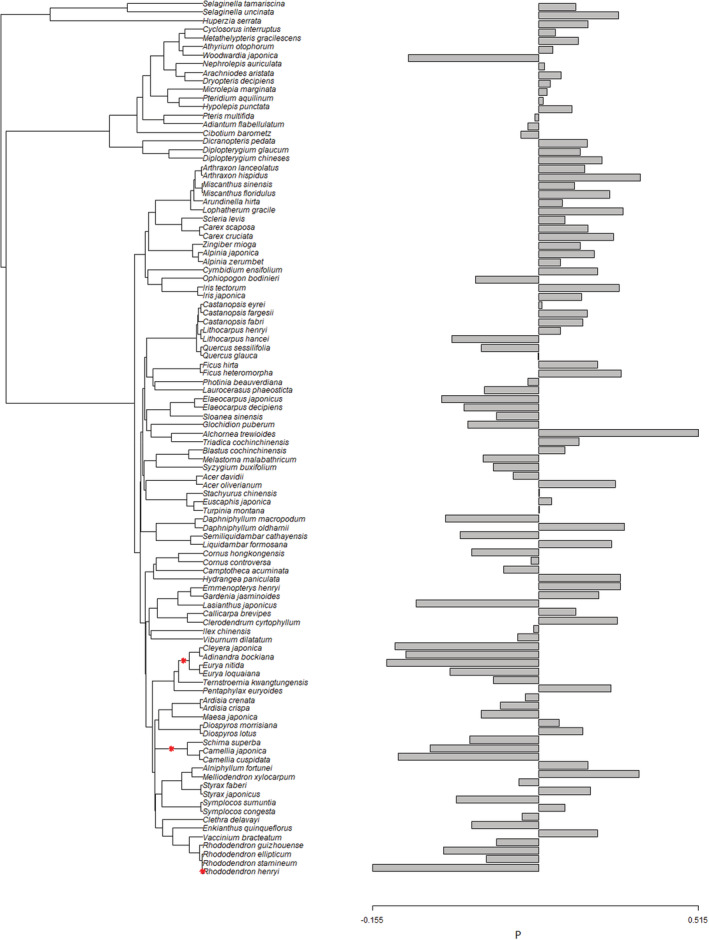
The positions (red stars) of the selective optima of leaf P concentration in the phylogeny.

**FIGURE A5:**
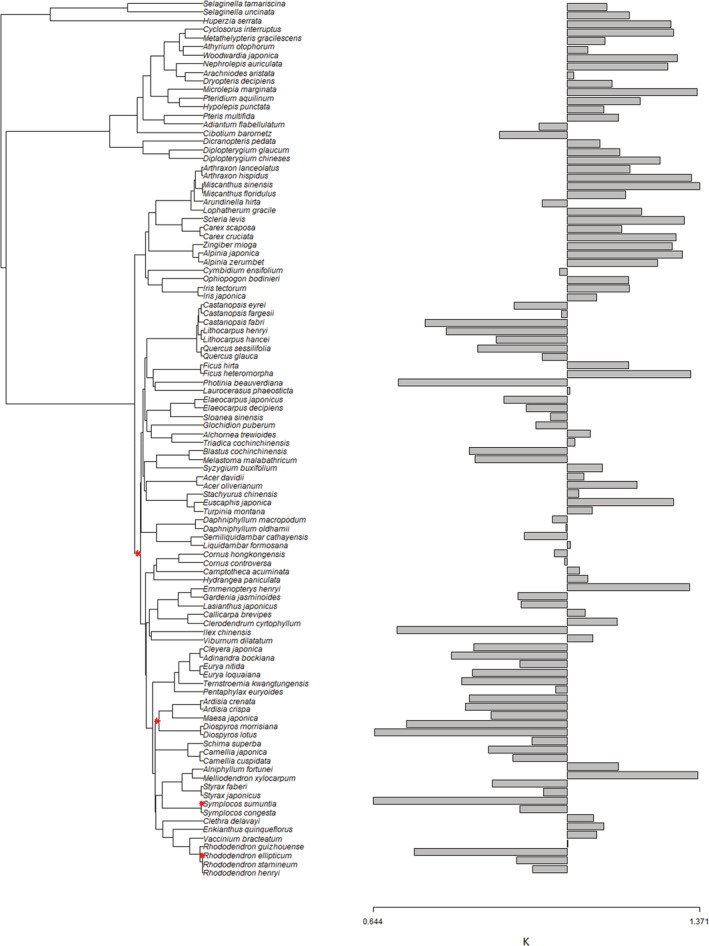
The positions (red stars) of the selective optima of leaf K concentration in the phylogeny.

**FIGURE A6:**
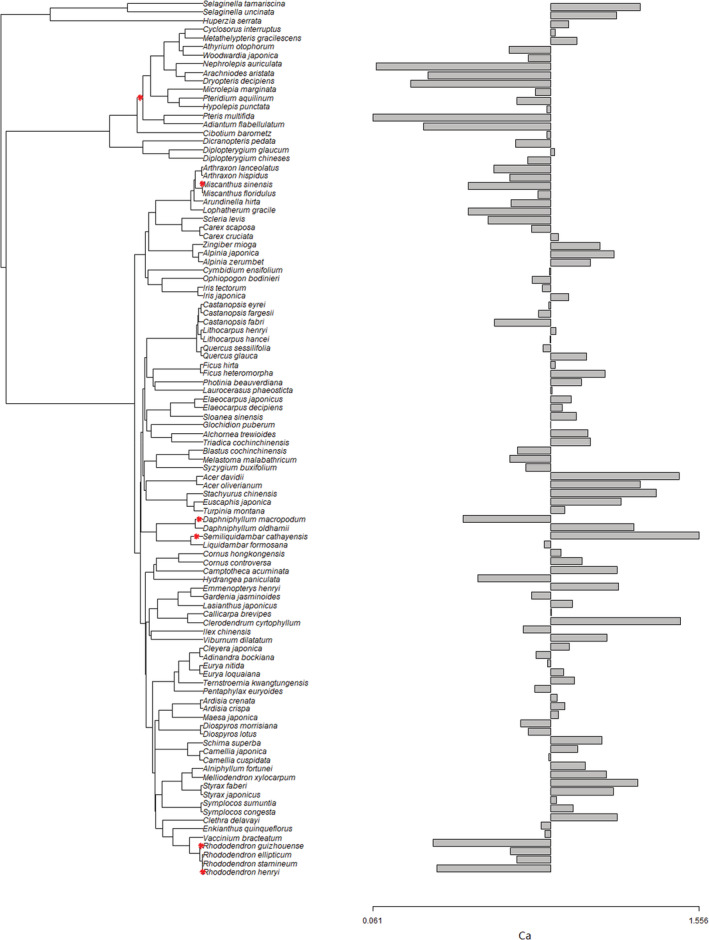
The positions (red stars) of the selective optima of leaf Ca concentration in the phylogeny.

**FIGURE A7:**
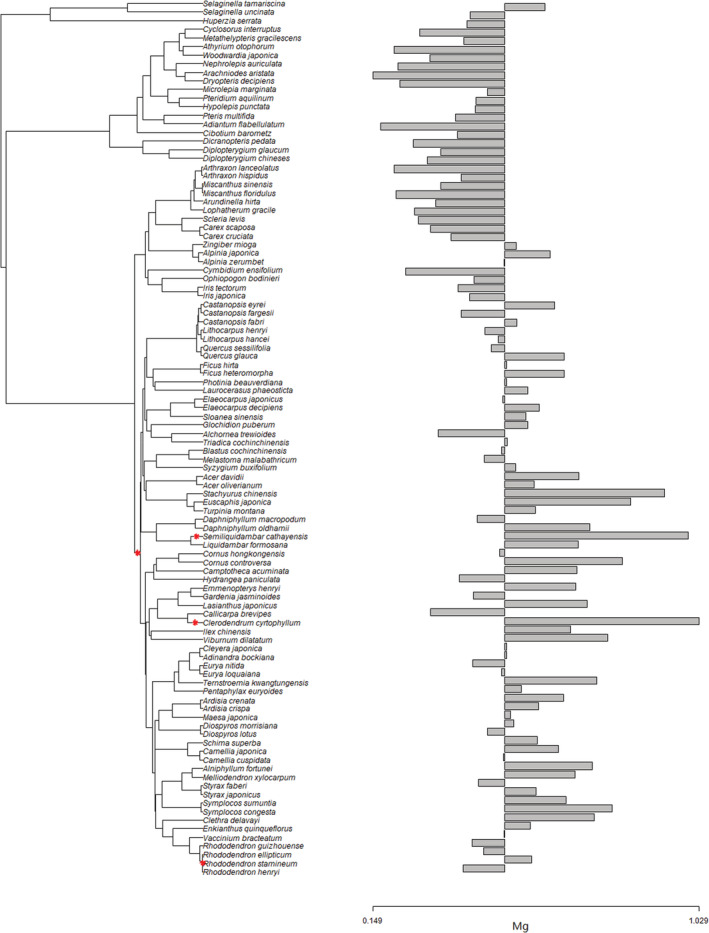
The positions (red stars) of the selective optima of leaf Mg concentration in the phylogeny.

The number of optima for leaf elemental concentrations in the phylogeny differed among elements. Leaf C concentration had only one optimum that was selected at the node toward the group of three fern species (i.e., *H. serrata*, *S. tamariscina*, and *S. uncinata*; Figure A2 in Appendix). Leaf N concentration had three optima that were selected at the two tips (i.e., deciduous *Alchomea trewioides* and *Clerodendrum cyrtophyllum*) and the node towards the group of 12 herbs such as *Arthraxon hispidus* and *Alpinia japonica* (Figure A3 in Appendix). Leaf P concentration had three optima that were selected at the tip (i.e., evergreen *R. henryi*) and two nodes toward the group of three evergreen Theaceae species (i.e., *Schima superba*, *Camellia cuspidata*, and *C. japonica*) and the group of four evergreen Pentaphylaceae species such as *Cleyera japonica* and *Eurya nitida* (Figure A4 in Appendix). Leaf K concentration had four optima that were selected at two tips (i.e., evergreen *R. ellipticum* and *Symplocos sumuntia*), the node toward the group of five woody species such as *Maesa japonica* and the node towards the group of all woody species (Figure A5 in Appendix). Leaf Ca concentration had six optima that were selected at five tips (i.e., *R. henryi*, *R. guizhouense*, *D. macropodum*, *Semiliquidambar cathayensis*, and *Miscanthus sinensis*) and the node towards the group of 12 Polypodiales ferns such as *Pteris multifida* and *N. aurivulata*) (Figure A6 in Appendix). Leaf Mg concentration had four optima that were selected at three tips (i.e., *R. stamineum*, *S. cathayensis*, and *C. cyrtophyllum*) and the node towards the group of all woody species (Figure A7 in Appendix). For PC1 loaded with leaf C, N, P, and K concentrations, only one optimum was selected at the node toward the group of all woody species. For PC2 loaded with leaf Ca and Mg concentrations, eight optima were selected at three tips (i.e., *R. henryi*, *S. cathayensi*, and *C. cyrtophyllum*), four nodes within herbaceous species (e.g., the node towards the group of 16 Polypodiales ferns), and one node within woody species (i.e., the node toward the group of five deciduous species such as *Acer dividii* and *Turpinia montana* (Figure 3).

Leaf C concentration was significantly and negatively correlated with leaf N, P, and K concentrations in all species (Table 3). Leaf Ca and Mg concentrations were significantly and positively correlated, as did for the correlations among leaf N, P, and K concentrations (Table 3).

**TABLE 3:** Phylogenetic generalized least squares regression analyses for the correlations between leaf elemental concentrations in all species.

	C	N	P	K	Ca	Mg
C						
N	−0.14***					
P	−0.09***	0.44***				
K	−0.08***	0.26***	0.36***			
Ca	−0.01	0.03	0.07	0.09		
Mg	−0.02	0.01	0.01	0.16	0.97***	

*Note*: The slope estimates and significant levels (***p < .001, **p < .01, and *p < .05) are shown.

## 4 DISCUSSION

### 4.1 Evidence of biogeochemical niche differences in plants

We found large means and ranges of leaf elemental composition across all species (Table 1), providing evidence of species‐specific biogeochemical niche differences in the subtropical montane evergreen broad‐leaved forest. Mean concentrations of the six elements were in descending order: C > N > K > Ca > Mg > P (Table 1), which almost agrees with the rankings of botanical garden plants and global forest plants (Peñuelas et al., 2019; Watanabe & Azuma, 2021). The ranges of variation in leaf elemental composition were lowest in C and highest in Ca (Table A2 in Appendix), similar to global forest plants (Peñuelas et al., 2019). These different means and ranges of leaf elemental composition suggest that the amount of each element required for maintaining plant growth and completing their life cycles varies greatly among plant species. The differential biogeochemical niches among species have also been found in bryophytes, ferns, herbs, and woody species (Amatangelo & Vitousek, 2008; Fernández‐Martínez et al., 2021; Sardans et al., 2021; Urbina et al., 2017). The observed biogeochemical niche differences among species in our study and previous studies reflect the different adaptations to absorb and utilize elements and thus can be considered as a mechanism maintaining species diversity in a given environment (Peñuelas et al., 2019; Silvertown, 2004).

We found that herbaceous and woody species had contrasting biogeochemical niches, as supported by their significantly different leaf elemental composition (Table 1). Herbaceous species in our study had significantly lower leaf C, Ca, and Mg concentrations but higher leaf N, P, and K concentrations than woody species, which is consistent with the research on savanna plants and global plants (Rossatto & Franco, 2017; Vergutz et al., 2012). This might be explained by the fact that woody species had thicker cell walls than herbaceous species, leading to higher leaf mass per area and the ensuing lower leaf N, P, and K concentrations (de la Riva et al., 2018; Onoda et al., 2017). By contrast, Watanabe and Azuma (2021) have demonstrated that herbaceous species had higher leaf Ca and Mg concentrations than woody species in a botanical garden. The discrepancies of leaf Ca and Mg concentrations between our study and their study might be due to the fact that herbaceous species in our study were mainly from Poales and Polypodiales, both of which tend to have lower leaf Ca and Mg concentrations (Funk & Amatangelo, 2013; Neugebauer et al., 2018). Like previous studies (e.g., Bai et al., 2019; de la Riva et al., 2018), we found that evergreen woody species had significantly higher leaf C concentration but lower leaf N, P, K, Ca, and Mg concentrations than deciduous woody species, suggesting the contrasting biogeochemical niches between leaf habits. This is largely due to the longer leaf life span in evergreen species that relates to larger construction cost and lower concentrations of mineral such as N, P, K, Ca, and Mg in leaves (Villar et al., 2006; Xing et al., 2021). We found that the differences of leaf elemental composition between ferns and herbs were element‐specific (Table 1). Leaf C, K, Ca, and Mg concentrations differed little between ferns and herbs. However, ferns had significantly lower leaf N and P concentrations than herbs in our study, which is consistent with observations on terrestrial plant species in China (Han et al., 2005). This could be due to the strong mesophyll limitation in ferns that can reduce the requirements of N and P for photosynthesis (Tosens et al., 2016).

The observed biogeochemical niche differences in subtropical forest plants can reflect their contrasting adaptations to the growth environmental factors such light, soil, and climate. The contrasting biogeochemical niche differences between herbaceous and woody species clearly reflect functional strategies regarding growth under different light conditions. Herbaceous species are usually concentrated on the forest floor where light availability is strongly suppressed (Detto et al., 2022). Poorter et al. (2019) have found that light availability is positively correlated with leaf C concentration but negatively correlated with leaf N and P concentrations across a wide range of plant species. Consistent with this finding, we detected the significantly higher concentrations of N and P concentrations in herbaceous species (Table 1), which could be a mechanism to increase carbon gain in the light‐limited forest floor (Poorter et al., 2019). In contrast to herbaceous species, woody species grow in relatively light‐rich environments such as sub‐canopy and canopy layers where light might not be a limiting factor for plant growth. Woody species might thus develop distinct functional strategies to cope with soil and climatic factors that might constrain leaf elemental composition. We found that leaf N, K, Ca and Mg concentrations in woody species were larger than the physiological concentration requirements (Kirkby, 2012) and bordered the sufficient concentration ranges for healthy growth (White & Brown, 2010), suggesting that N, K, Ca, and Mg might be sufficient for plant growth in our study site. However, leaf P concentration in woody species was lower than the physiological concentration requirement for P, suggesting that P might be a limiting element for plant growth. This is consistent with the general observation of P limitation in subtropical forests (Wen et al., 2018). The P limitation, together with subtropical hot and humid climate, could facilitate evergreen woody species to develop thicker and long‐lived leaves with lower mineral concentration requirements to adapt these soil and climatic conditions (Ye et al., 2022).

### 4.2 Evidence of biogeochemical niche conservatism in plants

Apart from adaptations to the environmental factors, biogeochemical niches in plants can be a legacy from their ancestors, which is referred as phylogenetic conservatism (Prinzing et al., 2001). We found that leaf C, N, K, and Mg concentrations had significant phylogenetic signals in all species (Table 2), suggesting the similarity of these leaf elements with increasing phylogenetic relatedness during species diversification in subtropical forest and leading support to biogeochemical niche conservatism. It is noteworthy that leaf C and K concentrations displayed the strongest phylogenetic signals in subtropical plants (Table 2). This agrees well with the observations that leaf C and K concentrations had highest Blomberg's *K* values across 119 herbaceous and woody species in a mosaic of temperate deciduous old‐growth forest and disturbed forest patches (Duffin et al., 2019). In contrast, we found that leaf Ca and P concentrations showed non‐significant phylogenetic signals in subtropical forest plants (Table 2), suggesting that phylogenetic relatedness might be less informative for the variations in the two leaf elements in subtropical forest plants. This is consistent with the detection of weak phylogenetic signal in leaf Ca and P concentrations in 58 topical lowland forest tree species (Metali et al., 2012) and in 40 tropical karst forest tree species (Bai et al., 2019). Our replicated phylogenetic signal tests on 100 randoms trees again revealed that phylogenetic conservatism was observed to be generally higher in leaf C, N, K, and Mg concentrations than leaf P and Ca concentrations (Table A1 in Appendix). We consider that these contrasting strengths of biogeochemical niche conservatism might be generated by different evolutionary processes.

The generation of biogeochemical niche conservatism was least described by BM model, because the highest AICc values were found in BM model for all leaf elements (Table 2). The weak possibility of BM model suggests that the evolution of biogeochemical niche was not likely to follow a neutral process. The values of Blomberg's *K* lower than unity in leaf elemental composition also revealed the weak possibility of BM model, against the drift and niche retention models (Cooper et al., 2010). Similarly, Bai et al. (2019) have demonstrated that the evolution of leaf concentrations of many nutrients (i.e., N, P, K, Ca, Mg, S, Fe, Cu, and Zn) could not be a pure BM process. These results suggest that biogeochemical niche might not change without limit through time partly because extreme values of leaf elemental composition (e.g., leaf elemental concentrations close to zero or infinity) are not biologically possible (Bai et al., 2019; Pan et al., 2014). Although BM model was not an effective model to describe the changes in leaf elemental composition along the phylogeny, it still can provide the information on the contrasting evolutionary rates among elements and between life forms. For instance, the sigma values were higher in leaf P and Ca concentrations and in woody species (Table 2), leading support to the evolutionary rate model and providing evidence of the less biogeochemical niche conservatism in the two elements and in woody species (Cooper et al., 2010; Münkemüller et al., 2015).

We found that Pagel's δ model was better than BM model and the δ values were higher than unity in all species (Table 3), which agrees with niche shifting model and indicates that the changes in biogeochemical niche in subtropical forest plants could expand to the present (Cooper et al., 2010; Pagel, 1999). The niche shifting model has also been detected to outperform BM model in the evolution of leaf elemental concentrations in tropical karst seasonal rainforest tree species (Bai et al., 2019). Under the niche shifting model, the biogeochemical niches of modern species might shift into a different portion of niche space as a consequence of selection to exploit new elemental resources, suggesting the species‐specific adaptations to present environment (Cooper et al., 2010; Hernández et al., 2013). It's noteworthy that δ value was largest in leaf P concentration, suggesting that the speed of trait trend expanding to the present was fastest in leaf P concentration. We considered that the fastest speed of trait trend expanding to the present could facilitate the species‐specific adaptations to changes in P resources in local environments.

We found that OU1 model also outperformed BM model for all leaf elements (Table 2), supporting the phylogenetic inertia model for the evolution of biogeochemical niche in subtropical forest plants (Cooper et al., 2010). OU1 model has also been found to outperform BM model for the evolution of leaf elemental concentrations in local and global plants (e.g., Bai et al., 2019; Fernández‐Martínez et al., 2018). Under OU1 model, the phylogenetic inertia (i.e., ɑ) could make leaf elemental concentrations to stabilize around the optimal composition, which could act as an evolutionary filter to exclude the species with unfit leaf elemental composition. For example, evergreen woody species with high leaf N and P concentrations might be excluded because this leaf elemental composition is not adaptive in nutrient‐limited subtropical forest. We found that the ɑ value was largest in leaf P concentration (Table 3), which suggests that plant species might have the strongest phylogenetic inertia to maintain optimal leaf P concentration in subtropical forest.

We found that OUM model might be the best model to generate biogeochemical niche conservatism due to its lowest AICc values in all leaf elements (Table 2). We found that leaf elemental concentrations could evolve around the distinct optimal values between herbaceous and woody species, as shown by higher Opt_H_ than Opt_W_ for leaf N, P, and K concentrations (Table 2). The higher optimal concentrations of the three elements in herbaceous species might be evolutionarily selected to widen the stomata openness (through K) for maximizing photosynthesis (through N and P), facilitating their growth and survival in light‐limited forest floor (Rawat et al., 2022; Wang et al., 2013). By contrast, the higher optimal concentrations of leaf C, Ca, and Mg concentrations were selected in woody species (Table 2), leading to the greater power to maintain the stability of cell wall chemistry in leaves (Neugebauer et al., 2018; White et al., 2018).

We found that the number of optima under an OU process throughout the phylogeny varied from one for C to six for Ca, suggesting the different number of optima among leaf elements (Figures A2, A3, A4, A5, A6, A7 in Appendix). According to a simulation study on trait conservatism, the increasing number of optima tends to decrease phylogenetic signal (Münkemüller et al., 2015). Consistent with this finding, leaf C concentration with the least optima presented the strongest phylogenetic signal while leaf Ca concentration with the most optima exhibited non‐significant phylogenetic signal (Table 2). This might be due to the fact that the more optima could increase the frequency of convergence, which could drive distantly related species towards the same optimal leaf elemental composition (Arbuckle et al., 2014). We found that the positions of optima could also vary among leaf elements. Some optima were concentrated towards the tips of phylogeny. For instance, one of the optima for leaf N and Mg concentrations were selected at the tip *C. cyrtophyllum* presenting high concentration values of the two elements. In contrast, the tips *R. henryi*, *S. sumuntia, and D. macropodum* were selected as an optimum towards low values of leaf P, K, and Ca concentrations, respectively. The positions of optima at the tips suggest the species‐specific adaptations to the local element resources and could make the tips showing distinct values of leaf elemental composition comparable to their closely related sisters, which could erase the biogeochemical niche conservatism in the phylogeny (Saccone et al., 2017). We found that the optima of leaf elemental composition were also selected at the nodes representing different clades within woody or herbaceous species (Figures A2, A3, A4, A5, A6, A7 in Appendix), providing information on biogeochemical niche conservatism at the clade level (Münkemüller et al., 2015). For instance, the node towards the group of 12 herbs such as *A. hispidus* and *A. japonica* was selected as an optimum towards high values of leaf N concentration. Similarly, optima toward low values of leaf P and Ca concentrations were selected at the nodes towards the group of four evergreen species such as *C. japonica* and *E. nitida* and the group of 12 Polypodiales ferns such as *P. multifida* and *N. auriculata*, respectively. These selected optima at the clade levels suggest that distantly related species within the clade tended to have similar leaf elemental concentrations, erasing the phylogenetic signal at the clade level. In addition, we found that the optima towards low levels of leaf K and Mg could be selected at the node towards the group of all woody species. This means that there might be the strong convergent evolution of the two leaf elements in woody species, making distantly related species to show similar concentrations of the two leaf elements. Collectively, our results show that the number and positions of optima were important predicators of biogeochemical niche conservatism in subtropical forest plants.

We found differential interaction strengths among leaf elements (Table 3). Specifically, leaf C, N and P and K concentrations were strongly inter‐correlated but weakly correlated with leaf concentrations of Ca and Mg across all species. Our results are similar to previous studies that have identified a metabolically active nucleic acid–protein set of leaf elements such as N, P, S, and Fe distinct from structural and enzymatic sets of leaf elements such as Ca, Mg, Mn, and Zn among plant species at local, regional and global scales (de la Riva et al., 2018; Watanabe et al., 2007; Zhang et al., 2012). The strong correlations among leaf elements across species might suggest that there is a phylogenetic effect governing the coordinated evolution among leaf elements (Bai et al., 2020; Donovan et al., 2011). For instance, leaf N and P concentrations have been found to be strongly and positively correlated through the phylogeny in African woody savanna species, indicating that a significant historical relationship between the two elements exists throughout the evolutionary history of this savanna plant group (Wigley et al., 2016). In addition, we found that both PC1 loaded with leaf C, N, P, and K concentrations and PC2 loaded with leaf Ca and Mg concentration presented significant phylogenetic signals (Table 2). This is similar to the finding of the significant phylogenetic signals in the scores of the first six principal component analysis axes derived from leaf N, P, K, S, Ca, and Mg concentrations and their ratios in global forest trees (Sardans et al., 2021). These findings of strong phylogenetic signals in PCA axes in our and other studies reinforce the evidence for the generally similar biogeochemical niches in closely related species. Therefore, the strong interactions among leaf elementals reflect the natural selection by favoring species with combinations of leaf elements that function well in a given environment and can serve as important agents of biogeochemical niche conservatism.

## 5 CONCLUSION

Our results demonstrate that the means and ranges of leaf elemental composition varied greatly in all species, suggestive of species‐specific biogeochemical niche in subtropical forest plants. We found that leaf elemental composition differed significantly between life forms (i.e., herbaceous/woody species, evergreen/deciduous woody species), suggesting the important role of life form in biogeochemical niche variation. We found that phylogenetic signal was significant in leaf C, N, K, and Mg concentrations but non‐significant in leaf P and Ca concentrations, which could be partly explained by the lower values of evolutionary rate (sigma), niche shifting strength (Pagel's δ) and phylogenetic inertia (α) in the former elements. However, the contrasting strengths of biogeochemical niche conservatism among elements were best generated by OU processes toward optima. We found that herbaceous species were evolutionarily selected to have higher optimal leaf N, P, K concentrations and lower optimal leaf C, Ca, and Mg concentrations than woody species, suggesting the different optima for the two life forms. We found the number of optima varied from the least in leaf C concentration to the most in leaf Ca concentration, suggesting that leaf Ca concentration might experience stronger convergent evolution in the phylogeny. Furthermore, we found the positions of optima were distributed unevenly within herbaceous and woody species in the phylogeny. The positions of optima towards the tips were more located in woody species, suggesting the more frequency of species‐specific adaptations in woody species. The positions of optima were also selected at the nodes towards the species grouping from certain life forms. For instance, the group of 12 Polypodiales ferns was converged to have similar low leaf Ca concentration and the group of three evergreen Theaceae species was converged to present similar low leaf P concentration. In addition, we found strong correlations among leaf C, N, P, and K concentrations and between leaf Ca and Mg concentrations, suggesting the coordinated evolution among elements. Our results thus show that biogeochemical niche conservatism relates to plant species diversification and life form evolution in subtropical forest plants. Furthermore, the strengths of biogeochemical niche conservatism varying among elements and between life forms might be mostly determined by the tempo and mode of OU processes.

## AUTHOR CONTRIBUTIONS

**Kundong Bai:** Conceptualization (lead); data curation (lead); formal analysis (lead); funding acquisition (lead); investigation (lead); methodology (lead); project administration (lead); writing – original draft (lead). **Xuewen Zhou:** Investigation (supporting); methodology (supporting); writing – original draft (supporting). **Shihong Lv:** Investigation (supporting); writing – original draft (supporting). **Shiguang Wei:** Formal analysis (supporting); writing – original draft (supporting). **Lili Deng:** Funding acquisition (supporting); investigation (supporting); writing – original draft (supporting). **Yibo Tan:** Investigation (supporting); writing – original draft (supporting).

## CONFLICT OF INTEREST

The authors have no conflict of interest to declare.

## Data Availability

Data from this study are available in Table A2 in Appendix.

## Appendix

**TABLE A1:** Summary of phylogenetic signal tests for leaf elemental concentrations and species scores of the first two principal component analysis axes (PC1 and PC2) on100 random trees.

	Significant signal	Non‐significant signal
C	96	4
N	100	0
P	3	97
K	92	8
Ca	2	98
Mg	81	19
PC1	100	0
PC2	80	20

*Note*: The frequencies (%) of significant and non‐significant phylogenetic signals are shown.

**TABLE A2:** Leaf elemental concentrations (mg g^−1^) in subtropical forest plants.

Species	Family	Life form	C	N	P	K	Ca	Mg
*Adinandra bockiana*	Pentaphylacaceae	Evergreen woody	412	18.56	0.82	6.58	6.45	3.23
*Ardisia crenata*	Primulaceae	Evergreen woody	459	16.81	1.44	7.21	8.02	4.61
*Ardisia crispa*	Primulaceae	Evergreen woody	449	17.23	1.28	7.08	8.72	3.95
*Blastus cochinchinensis*	Melastomataceae	Evergreen woody	438	25.82	1.74	7.23	5.28	3.13
*Callicarpa brevipes*	Lamiaceae	Evergreen woody	404	25.32	1.83	13.08	7.58	2.02
*Camellia cuspidata*	Theaceae	Evergreen woody	433	17.41	0.79	9.01	7.35	3.17
*Camellia japonica*	Theaceae	Evergreen woody	406	19.30	0.92	7.98	10	4.46
*Castanopsis eyrei*	Fagaceae	Evergreen woody	421	19.22	1.56	9.08	7.36	4.35
*Castanopsis fabri*	Fagaceae	Evergreen woody	467	20.21	1.89	5.76	4.15	3.45
*Castanopsis fargesii*	Fagaceae	Evergreen woody	412	22.52	1.93	11.58	6.62	2.44
*Cleyera japonica*	Pentaphylacaceae	Evergreen woody	456	16.31	0.78	7.40	9.15	3.23
*Cornus hongkongensis*	Cornaceae	Evergreen woody	432	19.23	1.12	11.16	8.38	3.10
*Daphniphyllum macropodum*	Daphniphyllaceae	Evergreen woody	413	17.46	0.99	11.07	2.99	2.70
*Daphniphyllum oldhamii*	Daphniphyllaceae	Evergreen woody	441	17.20	2.30	11.86	18.1	5.41
*Elaeocarpus decipiens*	Elaeocarpaceae	Evergreen woody	437	17.90	1.08	9.66	8.48	3.96
*Elaeocarpus japonicus*	Elaeocarpaceae	Evergreen woody	483	18.66	0.97	8.62	9.36	3.16
*Eurya loquaiana*	Pentaphylacaceae	Evergreen woody	432	15.65	1.01	7.33	8.61	3.14
*Eurya nitida*	Pentaphylacaceae	Evergreen woody	462	17.76	0.75	9.35	7.25	2.62
*Gardenia jasminoides*	Rubiaceae	Evergreen woody	422	24.56	2.04	9.27	6.13	2.63
*Glochidion puberum*	Phyllanthaceae	Evergreen woody	407	16.88	1.10	10.17	7.54	3.69
*Ilex chinensis*	Aquifoliaceae	Evergreen woody	428	18.03	1.5	4.99	5.63	4.81
*Lasianthus japonicus*	Rubiaceae	Evergreen woody	418	18.88	0.86	9.43	9.44	5.33
*Laurocerasus phaeosticta*	Rosaceae	Evergreen woody	431	19.82	1.19	12.08	7.61	3.68
*Lithocarpus hancei*	Fagaceae	Evergreen woody	498	19.19	1.02	8.28	7.49	3.07
*Lithocarpus henryi*	Fagaceae	Evergreen woody	435	20.48	1.70	6.41	7.92	2.83
*Maesa japonica*	Primulaceae	Evergreen woody	405	15.23	1.17	8.08	8.16	3.31
*Melastoma malabathricum*	Melastomataceae	Evergreen woody	427	14.38	1.18	7.43	4.88	2.82
*Pentaphylax euryoides*	Pentaphylacaceae	Evergreen woody	425	18.82	2.16	11.23	6.37	3.54
*Quercus glauca*	Fagaceae	Evergreen woody	451	22.20	1.53	10.48	10.97	4.62
*Quercus sessilifolia*	Fagaceae	Evergreen woody	447	19.20	1.17	7.53	6.92	2.94
*Rhododendron ellipticum*	Ericaceae	Evergreen woody	476	15.21	0.98	5.45	4.91	2.81
*Rhododendron guizhouense*	Ericaceae	Evergreen woody	420	22.23	1.26	11.96	2.18	2.61
*Rhododendron henryi*	Ericaceae	Evergreen woody	458	15.32	0.70	9.98	2.27	2.47
*Rhododendron stamineum*	Ericaceae	Evergreen woody	445	18.51	1.20	9.19	5.25	3.77
*Schima superba*	Theaceae	Evergreen woody	427	17.87	1.11	9.95	12.9	3.92
*Semiliquidambar cathayensis*	Altingiaceae	Evergreen woody	409	17.38	1.06	9.57	36	9.99
*Sloanea sinensis*	Elaeocarpaceae	Evergreen woody	394	27.24	1.26	10.94	9.83	3.64
*Styrax faberi*	Styracaceae	Evergreen woody	450	18.71	1.4	8.12	18.8	2.72
*Symplocos congesta*	Symplocaceae	Evergreen woody	413	22.88	1.74	9.36	9.52	6.23
*Symplocos sumuntia*	Symplocaceae	Evergreen woody	425	17.29	1.04	4.41	8.01	4.68
*Syzygium buxifolium*	Myrtaceae	Evergreen woody	406	17.33	1.24	14.30	5.78	3.42
*Ternstroemia kwangtungensis*	Pentaphylacaceae	Evergreen woody	440	20.78	1.24	6.95	9.68	5.65
*Vaccinium bracteatum*	Ericaceae	Evergreen woody	405	23.03	2.03	13.87	7.06	3.18
*Acer davidii*	Sapindaceae	Deciduous woody	420	20.47	1.36	13.00	29.1	5.06
*Acer oliverianum*	Sapindaceae	Deciduous woody	402	22.38	2.21	17.05	19.32	3.83
*Alchornea trewioides*	Euphorbiaceae	Deciduous woody	419	38.18	3.27	13.41	11.09	2.12
*Alniphyllum fortunei*	Styracaceae	Deciduous woody	418	23.33	1.94	15.48	10.82	5.50
*Camptotheca acuminata*	Nyssaceae	Deciduous woody	372	22.04	1.30	12.67	15.12	5.01
*Clerodendrum cyrtophyllum*	Lamiaceae	Deciduous woody	404	36.82	2.23	15.41	29.5	10.68
*Clethra delavayi*	Clethraceae	Deciduous woody	413	20.41	1.42	13.64	15.2	5.57
*Cornus controversa*	Cornaceae	Deciduous woody	399	21.33	1.48	11.78	10.45	6.64
*Diospyros lotus*	Ebenaceae	Deciduous woody	409	20.25	1.89	4.45	5.93	2.87
*Diospyros morrisiana*	Ebenaceae	Deciduous woody	389	23.76	1.69	5.23	5.48	3.38
*Emmenopterys henryi*	Rubiaceae	Deciduous woody	364	19.69	2.26	22.30	15.34	4.97
*Enkianthus quinqueflorus*	Ericaceae	Deciduous woody	418	18.91	1.12	14.39	6.79	3.75
*Euscaphis japonica*	Staphyleaceae	Deciduous woody	400	27.53	1.63	20.52	15.82	6.98
*Ficus heteromorpha*	Moraceae	Deciduous woody	378	26.97	2.27	22.47	13.3	4.63
*Ficus hirta*	Moraceae	Deciduous woody	380	28.83	2.03	16.31	7.89	3.23
*Hydrangea paniculata*	Hydrangeaceae	Deciduous woody	382	23.00	2.26	13.26	3.48	2.41
*Liquidambar formosana*	Altingiaceae	Deciduous woody	391	28.92	2.17	12.13	7.03	5.05
*Melliodendron xylocarpum*	Styracaceae	Deciduous woody	381	29.03	2.47	23.27	13.52	4.94
*Photinia beauverdiana*	Rosaceae	Deciduous woody	405	23.73	1.46	5.02	10.38	3.24
*Stachyurus chinensis*	Stachyuraceae	Deciduous woody	374	20.41	1.54	12.63	22.85	8.62
*Styrax japonicus*	Styracaceae	Deciduous woody	392	28.70	1.96	10.57	14.55	3.88
*Triadica cochinchinensis*	Euphorbiaceae	Deciduous woody	426	23.71	1.86	12.41	11.43	3.25
*Turpinia montana*	Staphyleaceae	Deciduous woody	405	24.35	1.54	13.53	8.75	3.87
*Viburnum dilatatum*	Adoxaceae	Deciduous woody	410	17.34	1.39	13.60	13.57	6.05
*Alpinia japonica*	Zingiberaceae	Herb	410	28.67	2.00	21.52	14.65	4.23
*Alpinia zerumbet*	Zingiberaceae	Herb	395	27.56	1.70	18.95	11.42	3.19
*Arthraxon hispidus*	Poaceae	Herb	370	32.59	2.48	22.49	4.88	2.44
*Arthraxon lanceolatus*	Poaceae	Herb	387	30.23	1.91	16.43	4.12	1.61
*Arundinella hirta*	Poaceae	Herb	392	23.21	1.72	10.48	4.96	2.08
*Carex cruciata*	Cyperaceae	Herb	357	28.56	2.19	20.82	8.13	2.29
*Carex scaposa*	Cyperaceae	Herb	385	22.27	1.94	15.76	6.13	2.02
*Cymbidium ensifolium*	Orchidaceae	Herb	419	21.55	2.03	11.48	7.42	1.73
*Iris japonica*	Iridaceae	Herb	401	19.58	1.88	13.85	9.11	2.57
*Iris tectorum*	Iridaceae	Herb	369	27.72	2.25	16.41	6.87	2.39
*Lophatherum gracile*	Poaceae	Herb	382	28.65	2.29	17.43	3.16	1.83
*Miscanthus floridulus*	Poaceae	Herb	376	29.77	2.15	16.06	6.56	1.63
*Miscanthus sinensis*	Poaceae	Herb	380	31.83	1.82	23.52	3.14	2.15
*Ophiopogon bodinieri*	Asparagaceae	Herb	409	20.55	1.14	16.33	6.17	2.64
*Scleria levis*	Cyperaceae	Herb	394	23.29	1.74	21.69	3.89	1.87
*Zingiber mioga*	Zingiberaceae	Herb	421	28.37	1.87	20.38	12.65	3.43
*Adiantum flabellulatum*	Pteridaceae	Fern	455	15.62	1.46	10.32	1.97	1.48
*Arachniodes aristata*	Dryopteridaceae	Fern	407	22.78	1.71	12.34	2.06	1.41
*Athyrium otophorum*	Athyriaceae	Fern	427	19.67	1.64	13.23	4.84	1.61
*Cibotium barometz*	Cibotiaceae	Fern	367	16.70	1.41	8.43	7.2	2.38
*Cyclosorus interruptus*	Thelypteridaceae	Fern	391	25.21	1.66	20.53	7.87	1.89
*Dicranopteris pedata*	Gleicheniaceae	Fern	368	18.90	1.93	14.10	5.19	1.81
*Diplopterygium chineses*	Gleicheniaceae	Fern	397	20.32	2.07	19.19	5.89	1.98
*Diplopterygium glaucum*	Gleicheniaceae	Fern	401	17.81	1.87	15.58	7.85	2.15
*Dryopteris decipiens*	Dryopteridaceae	Fern	375	26.54	1.62	15.02	1.72	1.67
*Huperzia serrata*	Lycopodiaceae	Fern	337	28.25	1.94	20.29	9.06	2.53
*Hypolepis punctata*	Dennstaedtiaceae	Fern	443	25.69	1.80	14.38	7.24	2.66
*Metathelypteris gracilescens*	Thelypteridaceae	Fern	383	28.02	1.85	14.46	9.88	2.48
*Microlepia marginata*	Dennstaedtiaceae	Fern	414	26.12	1.60	23.19	6.38	2.87
*Nephrolepis auriculata*	Nephrolepidaceae	Fern	384	20.66	1.58	19.96	1.19	1.65
*Pteridium aquilinum*	Dennstaedtiaceae	Fern	438	27.11	1.57	17.32	5.27	2.67
*Pteris multifida*	Pteridaceae	Fern	425	21.60	1.51	15.50	1.15	2.36
*Selaginella tamariscina*	Selaginellaceae	Fern	363	20.80	1.83	14.60	19.35	4.11
*Selaginella uncinata*	Selaginellaceae	Fern	308	30.22	2.24	16.40	15.02	2.58
*Woodwardia japonica*	Blechnaceae	Fern	434	14.41	0.83	20.93	5.94	2.01
		Mean	411	22.21	1.61	12.89	9.02	3.49
		Minimum	308	14.38	0.70	4.41	1.15	1.41
		Maximum	498	38.18	3.27	23.52	36.00	10.68
		Max/Min	1.6	2.7	4.7	5.3	31.3	7.6
